# Effect of different antioxidant additives in semen diluent on cryopreservability (−196°C) of buffalo semen

**DOI:** 10.14202/vetworld.2016.299-303

**Published:** 2016-03-20

**Authors:** Hardik A. Patel, G. M. Siddiquee, Dinesh V. Chaudhari, Vishal S. Suthar

**Affiliations:** 1 Sabarmati Ashram Gaushala, Ahmedabad, Gujarat, India; 2 Department of Gynaecology and Obstetrics, College of Veterinary Sceicne and Animal Husbandry, Sardarkrushinagar Dantiwada Agricultural University, Deesa, Banaskanth, Gujarat, India; 3 Pashupalan Sansodhan Kendra, Veterinary College, Anand Agricultural University, Anand, Gujarat, India; 4 Directorate of Research, Kamdhenu University, Gandhinagar, Gujarat, India

**Keywords:** antioxidants, cryopreservation, diluent, egg yolk, enzyme, semen

## Abstract

**Aim:**

The aim of this study was to evaluate the effect of different antioxidant additives in standard tris-fructose-egg yolk-glycerol (TFYG) extender on the cryopreservability of buffalo semen.

**Materials and Methods:**

Semen collection using artificial vagina, twice weekly for 5 weeks from three pedigreed health breeding bulls of Mehsani breed, aged between 6 and 8 years. Immediately after initial evaluation all 30 qualifying ejaculates (10/bull) were split into three aliquots and diluted at 34°C keeping the concentration of 100 million spermatozoa/ml with standard TFYG extender as control and TFYG having two antioxidant additives - Cysteine HCl at 1 mg/ml and ascorbic acid at 0.2 mg/ml to study their comparative performance. Semen filled in French Mini straws using IS-4 system and gradually cooled to 4°C and equilibrated for 4 h in cold handing cabinet. After completion of equilibration, straws were cryopreserved in LN_2_ by Programmable Bio-freezer. Semen was examined at post-dilution, post-equilibration, and post-thaw stages for sperm quality parameters, and at each stage plasma was separated for enzymatic analysis of aspartate aminotransferase (AST), lactate dehydrogenase (LDH), and alkaline phosphatase (AKP).

**Results:**

The mean percentage of sperms in TFYG, TFYG + cysteine HCl and TFYG + ascorbic acid diluents at post-thaw stage in terms of progressive motility (52.83±0.52, 57.83±0.52, 57.83±0.52), livability (78.70±0.21, 82.33±0.23, 81.73±0.22), and abnormality (5.43±0.21, 5.03±0.17, 5.23±0.18) varied significantly (p<0.05) between control TFYG and TFYG having antioxidant additives. The mean U/L activities of AST (78.70±0.47, 72.80±0.48, 73.30±0.54), LDH (172.70±0.41, 155.78±0.42, 156.33±0.41), and AKP (103.61±0.34, 90.20±0.34, 91.03±0.34) in semen diluted with TFYG, TFYG + cysteine HCl and TFYG + ascorbic acid diluents at post-thaw stage, respectively, which showed significantly (p<0.05) higher leakage of enzymes in control TFYG than TFYG incorporated with additives.

**Conclusion:**

Incorporation of antioxidant additives such as cysteine HCl and ascorbic acid in standard TFYG diluents improves sperm quality parameters, reduces enzyme leakage, and ultimately advances cryopreservability of buffalo semen.

## Introduction

Artificial insemination is one of the important procedures, which causes the widespread propagation of semen, limiting the spread of sexually transmitted diseases and chiefly facilitating genetic improvement programs. Poor keeping quality and freezability of buffalo semen is documented in the literature by many research workers [1,2]. It is suggested that higher susceptibility of mammalian spermatozoa toward oxidative stress may be due to higher lipid peroxidation levels. Although bovine semen has a natural defense system against the oxidative stress, it is considered insufficient under cryopreservation-mediated stress. The extender is an important factor in cryopreservation process which should has adequate pH and buffering capacity, appropriate osmolality and should protect spermatozoa from the cryogenic lesion. Reinforcement of semen extender with suitable additives is suggested to reduce oxidative damage during freeze-thawing of bull and buffalo spermatozoa [3].

Motility and fertilization ability of spermatozoa can be improved by addition of various motility enhancing agents or antioxidants. The antioxidants check the chemical breakdown of the substrate resulting from oxidation and neutralize the free radicals and reduce the risk of damage to spermatozoa during cryopreservation [4]. Amino acids have an important role in preventing oxidative damage to spermatozoa during preservation. Cysteine, a precursor of intracellular glutathione (GSH), has been shown to penetrate the cell membrane easily, enhancing the intracellular GSH biosynthesis both *in vivo* and *in vitro* and protecting the membrane lipids and proteins due to indirect radical scavenging properties. Cysteine has cryoprotective effect on the functional integrity of axosome and mitochondria improving post-thawed sperm motility in many species [5]. The seminal plasma contains about 65% of the antioxidant capacity in the form of ascorbic acid, hence showing the importance of this vitamin. Ascorbic acid concentration in seminal plasma exceeds 10 times more than that in blood plasma (364 compared with 40 µmol/L) [6]. It is now known that the ascorbic acid content of buffalo bull semen is significantly lower as compared to cow bull semen [7,8]. This might be the reason for poor *in vitro* preservability of buffalo bull semen.

The seminal enzymes play a crucial role in fertilization and are affected due to cryopreservation. The assessment of levels of certain enzymes, *viz*., transaminases, dehydrogenases, in the seminal plasma are very important in judging the preservability and fertilizing capacity of spermatozoa [9].

Keeping these facts in priority, the present investigation was aimed to evaluate the effect of antioxidant additives such as cysteine HCl an’d ascorbic acid in tris-fructose-egg yolk-glycerol (TFYG) diluent on the preservability of Mehsani buffalo semen.

## Materials and Methods

### Ethical approval

The experiment was conducted following all the code of ethics for animal experimentation with prior approval by the Institute’s Animal Ethics Committee.

### Animals and sampling

The study was undertaken on three sexually mature healthy pedigreed breeding bulls of Mehsani breed of buffaloes, aged 6-8 years at State Frozen Semen Production and Training Institute, GLDB in Patan, Gujarat (India). All these bulls were in good health and under veterinary care. The semen was collected regularly twice in a week using artificial vagina. About 10 ejaculates were studied from each bull (total 10 × 3 = 30 ejaculates) at weekly interval in a split-sample technique.

### Semen extender preparations

The standard protocols as per FAO (1979) for the preparation of TFYG extender were followed and fresh extenders were prepared just before collection. The standard TFYG extender includes 80 ml tris buffer (containing 2.42 g tris, 1.36 g citric acid, 1.00 g fructose and 6.40 ml glycerol in 80 ml of Milli-Q water), 20 ml fresh egg yolk and antibiotics (penicillin at 1000 IU/ml and streptomycin at 1000 µg/ml). Three aliquots of standard TFYG extender were made into different glass cylinders. In one aliquot cysteine at 1 mg/ml and in another ascorbic acid at 0.2 mg/ml of TFYG extender was incorporated. The third aliquot of TFYG was kept as non-added control. Finally, prepared extenders were kept in thermo-regulatory water bath at 34°C until used for extension.

### Semen preservation and evaluation

Immediately after collection the ejaculates were shifted in water bath at 34°C and evaluated for routine macroscopic and motility attributes. Only the ejaculates with >70% initial motility were further used for the study. The ejaculate was divided into three aliquots and immediately diluted with different extender preparations at 100 million sperms/ml. The French Mini straws were filled using automatic filling-sealing machine (IS4 system, IMV, France) after keeping a fraction of semen for post-dilution evaluation. The filled straws after equilibration period of 4-5 h at 4°C in Cold Handling Cabinet (IMV, France) were cryopreserved in LN2 using Programmable Bio-freezer (IMV, France) having standard freeze curve for bovine semen (from 4 to −10°C at 5°C/min, −10 to −100°C at 40°C/min and −100 to −140°C at 20°C/min) and immediately submerged in LN2 for storage at −196°C. The semen samples were evaluated at post-dilution, post-equilibration, and post-thaw stages for sperm quality parameters such as progressive motility (by phase contrast microscopy), livability, and morphology (by eosin-nigrosin staining) as per standard procedure. Seminal plasma was separated at all above stages and stored in refrigeration for enzymatic analysis of aspartate aminotransferase (AST), lactate dehydrogenase (LDH), and alkaline phosphatase (AKP).

### Statistical analysis

The data generated were analyzed statistically using ANOVA and critical different test or Duncan’s new multiple range test by employing IBM SPSS Statistics version 20.00 to know the variation between different levels of additives and periods of preservation.

## Results and Discussions

The comparative study on the effect of antioxidants (cysteine HCl and ascorbic acid) on cryopreservability (−196°C) of Mehsani buffalo semen was carried out at post-dilution, post-equilibration, and post-thaw stages. The results revealed that there was a significant difference (p<0.05) in quality parameters between semen diluents preparations at a particular stage of preservation and between different stages.

On comparison the individual motility and live sperm percentage of semen differed significantly (p<0.01) between different stages, but there was no significant difference in abnormal sperm percentage between fresh and frozen thawed semen and the values remained almost same for all the three groups of semen.

**Figure-1:**
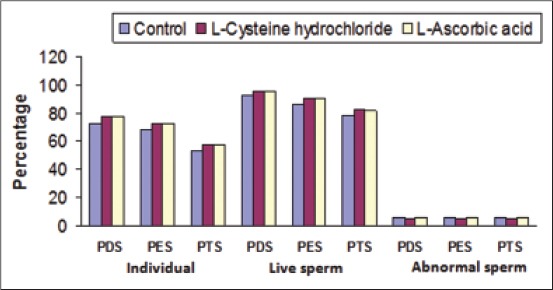
Individual motility percentage, live sperm percentage and abnormal sperm percentage at different stages of preservation with semen diluent additives.

The percentage of individual motile spermatozoa was significantly (p<0.05) higher at post-diluted, post-equilibrated, and post-thawed stages of semen preservation in cysteine HCl group (77.83±0.52, 72.83±0.52, and 57.83±0.52) and ascorbic acid group (77.83±0.52, 72.83±0.52, and 57.83±0.52) as compared to control group (72.83±0.52, 67.83±0.52, and 52.83±0.52). Similarly, the percentage of live sperm count was also significantly (p<0.05) higher at post-diluted, post-equilibrated, and post-thawed stages of semen preservation in cysteine HCl group (95.67±0.14, 90.43±0.20, and 82.33±0.23) and ascorbic acid group (95.63±0.13, 90.13±0.23, and 81.73±0.22) as compared to control group (93.20±0.19, 86.70±0.22, and 78.70±0.21). There was significantly (p<0.05) lesser abnormal sperm count in cysteine HCl group (5.03±0.17%) as compared to control group (5.43±0.21%) at all the stages of semen preservation but non-significant in ascorbic acid group (5.23±0.18%) as compared to the control group (5.43±0.21%) (Figure-1).

This result was similar to that reported by Hazarika *et al*. [10] in Murrah buffalo bulls who reported a significant difference in abnormal sperm count at 0 h post-freeze interval between cysteine HCl containing diluent and the control. Cysteine HCl has been added in TFYG extender, which improved the post-thaw Nili-Ravi buffalo bull spermatozoa functional assays [11]. The results indicate a higher progressive motility, plasma membrane, acrosomal integrities at 1 mM addition of cysteine HCl as compared to the control group [6] which was corroborated with our findings. Similarly, cysteine HCl in cryopreserved semen of Murrah buffalo bull found success in improving the quality features of spermatozoa [12]. However, this result was contrary to that reported by Virani [13] in Surti buffalo bulls who reported no significant difference in abnormal sperm count at any of the stages of semen preservation between cysteine HCl containing diluent and the control, but our findings revealed significant difference in sperm abnormality which was in line with the results of many others [3,6,11,14].

Shannon [15] demonstrated a factor, probably a protein in bovine seminal plasma which depressed livability and motility of the spermatozoa. The factor was heat labile and non-dialyzable. It had been suggested that the toxic effect of the factor might be due to its sulfydril binding capacity. A similar toxic factor had been demonstrated in buffalo seminal plasma [16]. This factor could be inactivated by addition of sulfydril group containing substances such as cysteine HCl. This was indicated in the present investigation by significant improvement in spermatozoal motility and viability in the presence of cysteine HCl. It had also been shown that aerobic fructolysis was stimulated in the presence of cysteine HCl and also the oxygen uptake by the spermatozoa was maintained for a longer time. This mechanism produced intensely reduced environment in the diluent media when semen was stored for a longer time [17].

The bovine spermatozoa motility significantly increases in extender supplemented with ascorbic acid at the concentration of 4.5 mg/ml [18]. The authors argue that 4.5 mg/ml enhances catalase activity, reduced GSH activity, and GSH peroxidase activity was significantly decreased. Perez and Perez [19] reported that a high ascorbic acid content was related to high sperm concentration and motility and a low percentage of abnormal sperm in HF bull and ram semen. Similarly, Stolbov and Rimanova [20] reported that addition of ascorbic acid to the egg yolk-lactose glycerol diluent, prior to freezing of bull semen improved the post-thaw sperm motility and survivability. Contrary to the present investigation, Virani [13] reported no significant differences in sperm motility and viability at the post-thaw stage of semen preservation between ascorbic acid containing diluent and the control. Increase in motility and viability of spermatozoa in the present investigation might be due to the antioxidant properties of ascorbic acid. Ascorbic acid due to its antioxidant properties prevents the toxic effect to spermatozoa which occur due to hydrogen peroxide release during the cryopreservation and hence the spermatozoa remain viable for a long period but not protected [17,21].

There were significant (p<0.05) differences in activities of AST, LDH and AKP enzymes among all stages in all three groups, i.e. control, cysteine HCl and ascorbic acid. The AST, LDH, and AKP levels were significantly (p<0.05) higher in ascending order at post-equilibrated and post-thawed stages as compared to post-diluted stage.

**Figure-2:**
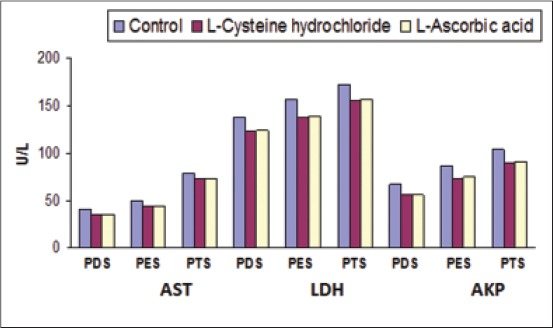
Leakage of aspartate aminotransferase (AST, U/L), lactate dehydrogenase (LDH, U/L) and alkaline phosphatase (AKP, U/L) from bull spermatozoa during different stages of preservation in diluents with and without additives.

Significantly (p<0.05) lower leakage of AST enzyme was noticed at post-diluted, post-equilibrated, and post-thawed stages 34.90±0.34, 44.33±0.27 and 72.80±0.48 U/L in cysteine HCl group and 35.20±0.35, 44.45±0.25 and 73.30±0.54 U/L in ascorbic acid group as compared to the control group 40.30±0.38, 49.82±0.29 and 78.70±0.47 U/L. There was significantly (p<0.05) lower leakage of LDH enzyme at post-diluted, post-equilibrated, and post-thawed stages (123.11±0.40, 138.43±0.34 and 155.78±0.42 U/L) in cysteine HCl group and (123.73±0.42, 138.85±0.29 and 156.33±0.41 U/L) in ascorbic acid group as compared to the control group (138.14±0.46, 157.38±0.36 and 172.70±0.41 U/L). The leakage of AKP enzyme was significantly (p<0.05) low at post-diluted, post-equilibrated, and post-thawed stages 55.56±0.35, 73.80±0.30 and 90.20±0.34 U/L in cysteine HCl group and 55.99±0.30, 74.33±0.27 and 91.03±0.34 U/L in ascorbic acid group as compared to the control group (67.07±0.32, 85.89±0.29 and 103.61±0.34 U/L) (Figure-2).

The enzyme leakages in fresh and frozen thawed semen in the present investigation were lower in diluents containing additives as compared to the control diluent. These results were in accordance with the report of Dhami and Sahni [12] and Khawaskar *et al*. [14] in buffalo bulls who reported that the enzyme leakage both at pre- and post-freeze was significantly greater in control diluent than those containing additives.

## Conclusions

There was an improvement in motility and viability of spermatozoa with lesser abnormality and reduced leakage of enzymes such as AST, LDH, and AKP in semen extended with diluents having cysteine HCl and ascorbic acid additives as compared to non-added control TFYG diluent. cysteine HCl was proved to be superior additive than ascorbic acid because it improved the sperm quality parameters in a better way at all the stages of semen preservation. Although supplementation of cysteine HCl and ascorbic acid in TFYG extender improved the quality parameters of cryopreserved Mehsani buffalo sperms, the routine use of such a supplementation in buffalo semen extender can be recommended after performing field fertility trials.

## Authors’ Contributions

This study is the part of M.V.Sc. thesis of the first author HAP, who carried out the research under the guidance of GMS. DVP helped during the trial. DVP and VSS helped in technical writing and revision of the article. All authors have read and approved the final version of the manuscript.
